# In-hospital and mid-term adverse clinical outcomes of a direct stenting strategy versus stenting after predilatation for the treatment of coronary artery lesions

**Published:** 2008-11

**Authors:** M Alidoosti, M Salarifar, SE Kassaian, AMH Zeinali, MS Fathollahi, MR Dehkordi

**Affiliations:** Department of Interventional Cardiology, Tehran Heart Centre, Medical Sciences, University of Tehran, Iran; Department of Interventional Cardiology, Tehran Heart Centre, Medical Sciences, University of Tehran, Iran; Department of Interventional Cardiology, Tehran Heart Centre, Medical Sciences, University of Tehran, Iran; Department of Interventional Cardiology, Tehran Heart Centre, Medical Sciences, University of Tehran, Iran; Department of Interventional Cardiology, Tehran Heart Centre, Medical Sciences, University of Tehran, Iran; Department of Interventional Cardiology, Tehran Heart Centre, Medical Sciences, University of Tehran, Iran

## Abstract

**Background:**

Direct stenting without balloon dilatation may reduce procedural costs and duration, and hypothetically, the restenosis rate. This study was designed to compare the in-hospital and long-term outcomes of direct stenting (DS) versus stenting after pre-dilatation (PS) in our routine clinical practice.

**Methods:**

The 1 603 patients treated with stenting for single coronary lesions were enrolled into a prospective registry. Patients with acute myocardial infarction (MI) within the preceding 48 hours, and those with highly calcified lesions, total occlusions, or a lesion in a saphenous graft were excluded. The baseline, angiographic and procedural data, inhospital outcomes and follow-up data were recorded in our database and analysed with appropriate statistical methods.

**Results:**

Eight hundred and fifty-seven patients (53.5%) were treated with DS and 746 (46.5%) underwent PS. In the DS group, lesions were shorter in length, larger in diameter and had lower pre-procedural diameter stenosis. Type C and diffuse lesions and drug-eluting stents were found less often (*p* < 0.001). With univariate analysis, dissection and non-Q-wave MI occurred less frequently in this group (0.2 and 0.6% vs 3.9 and 2.1%, *p* < 0.001 and *p* = 0.01, respectively). However, the cumulative major adverse cardiac events (MACE) did not differ significantly (4.9 vs 4.6%, *p* = 0.79). With multivariate analysis, direct stenting reduced the risk of dissection (OR = 0.07, 95% CI: 0.01–0.33, but neither the cumulative endpoint of MACE (OR = 1.1, 95% CI = 0.58–2.11, *p* = 0.7) nor its constructing components were different between the groups.

**Conclusions:**

Direct stenting in the real world has at least similar long-term outcomes in patients treated with stenting after pre-dilatation, and is associated with lower dissection rates.

## Summary

Since the advent of balloon angioplasty, the introduction of coronary stents has been the most important turning point in the percutaneous management of coronary artery lesions. Coronary stents are associated with more effective dilatation and predictable in-hospital outcomes, higher procedural success rates, and a decreased need for target-vessel revascularisation.^1^-^4^ Stents are now used in over 80% of percutaneous coronary interventions.^5^

The standard stent implantation technique requires routine pre-dilatation with a balloon catheter to allow an easy passage of the stent and to enhance a complete expansion of all stent modules.^6^ Therefore, there has been widespread use of stenting as an adjunct to plain balloon angioplasty in the setting of percutaneous coronary intervention. With the advance in stent and delivery system design and crimping, direct stenting without balloon pre-dilatation has become a feasible strategy in many catheterisation laboratories.^7^

The placement of stents without balloon dilatation may reduce the duration of the procedure, the radiation exposure, the amount of contrast media used, and the cost of the disposable supplies.^8^-^10^ Furthermore, by reducing the extent of vessel injury, direct stenting has been postulated to be relevant in reducing the restenosis rate.^11^,^12^ However, a number of disadvantages have been suggested for direct stenting, including failure to cross the lesion, incomplete stent deployment, an increase in guide trauma, undersizing the stent, and poor visualisation, which may result in errors in stent positioning.^13^

Animal models have shown that direct implant of a stent reduces the degree of intimal hyperplasia in comparison with prior balloon dilatation.^14^ However, randomised clinical trials have not proven the positive effect of direct stenting in reducing the restenosis rate. This study was designed to compare the in-hospital and long-term outcomes of direct stenting versus stenting after pre-dilatation in our routine clinical practice.

## Methods

Between March 2003 and 2005, 1 603 patients were enrolled in a prospective registry. The criterion for inclusion into the registry was the implantation of stents for single native coronary lesions with ≥ 50% stenosis in patients with no acute myocardial infarction (MI) within the preceding 48 hours. Patients with a highly calcified lesion, total occlusion, or a lesion in a saphenous graft were excluded from the study. The decision whether or not to pre-dilate was based on the attitudes of the operators. The mean age of participants was 55.96 ± 10.50 years (range: 25–88).

In this study, 857 patients (53.5%) were treated with stents without pre-dilatation (direct stenting), whereas 746 (46.5%) underwent stenting after balloon pre-dilatation. Baseline, clinical, angiographic and procedural characteristics, and in-hospital outcomes were obtained by research physicians and entered into a computerised database by computer operators. Finally, 88% of patients agreed to participate in follow-up programmes.

Clinical outcomes, most importantly, major adverse cardiac events (MACE) including cardiac death, non-fatal MI and target-vessel revascularisation [bypass surgery or repeated percutaneous coronary intervention (PCI)] were obtained by cardiologists in clinics at one, five and nine months post operation and once a year thereafter, or by formal telephone interviews, and recorded on data sheets, which were later entered into the computerised database.

This study was approved by the Tehran Heart Centre Ethics Committee. Informed consent was obtained from all patients before enrolment into this study.

## Coronary procedures

All angioplasty procedures were done with a number 6 or 7 French guiding catheter and a femoral approach. Patients received 600 mg of clopidogrel and 325 mg of aspirin before, and 7 500–10 000 IU of heparin at the start of the procedure. The femoral sheath was removed after normalisation (< 40 sec) of the partial thromboplastin time. Patients assigned to stenting after pre-dilatation (PS group) received one or more dilatations, followed by stent implantation to achieve a residual stenosis of less than 20%. The procedure was terminated if the residual stenosis was below 20% and no visible dissection with compromised flow was present.

In patients assigned to direct stent implantation (DS group), the target lesion was crossed with an appropriate stent without pre-dilatation. If crossing of the target lesion with the stent delivery system did not succeed, the patient was reallocated to the PS group. After stent placement, ticlopidine (250 mg twice daily) or clopidogrel (75 mg once daily) was given routinely for four weeks with bare metal stents and for six to 12 months with drug-eluting stents. Aspirin was given indefinitely to all patients.

Angiographic findings such as vessel dimensions, pre- and post-procedural stenoses, lesion length and thrombolysis in myocardial infarction (TIMI) flow grade were determined by visual estimation using the guiding catheter as a reference object for calibration. The angiographic characteristics were also further analysed by an independent interventional cardiologist not involved in the procedure and checked for inter-observer agreement.

## Definitions

Angina symptoms were defined according to the classification of the Canadian Cardiovascular Society.^15^ Lesion types were noted according to the American College of Cardiology/American Heart Association (ACC/AHA) lesion characteristics classification.^16^ Left ventricular ejection fraction was obtained from cardiac catheterisation ventriculograms. Q-wave MI was defined as the presence of new Q waves in post-procedure electrocardiograms, with a three-fold increase in the MB fraction of creatine kinase. Non-Q-wave MI was defined as a three-fold increase in the MB fraction of creatine kinase without the development of new Q waves.^17^

Angiographic success was defined as residual stenosis of less than 20% plus normal TIMI 3 flow. Procedural success was defined as angiographic success without major complications (death, MI, emergency bypass surgery or PCI) during hospitalisation. Major adverse cardiac events (MACE) were defined as the presence of cardiac death, non-fatal MI, or target-vessel revascularisation (TVR) during the follow-up period. Target vessel revascularisation was defined as ischaemia-driven repeat percutaneous intervention or bypass surgery of the target vessel. Target-lesion revascularisation (TLR) was defined as ischaemia-driven repeat percutaneous intervention of the target lesion or bypass surgery of the target vessel.^18^ Dissection was defined as the presence of angiographically apparent intimal or medial injury, according to the National Heart, Lung and Blood Institute criteria.^19^

The primary endpoint was to compare the occurrence of MACE between both groups during follow-up. As a secondary aim, we compared the rates of dissection and rising of cardiac enzymes (peri-procedural non-Q-wave MI) after stenting with both strategies, and determined their independent predictors.

## Statistical analysis

Statistical testing was performed by the chi-square test or Fisher’s exact test (two-tailed) for categorical variables. The Student’s *t*-test was used for comparison of continuous variables. Multivariate Cox proportional-hazards models using stepwise selection were developed for comparison of the rates of MACE and its constructing components (cardiac death, non-fatal MI and TVR) in follow-up. Event-free survival curves were drawn using the Kaplan-Meier method. The log-rank test was used to test for differences between survivals. The multivariate logistic regression model was constructed to determine the independent predictors of dissection and non-Q-wave MI in both groups.

Results are presented as adjusted odds ratios with 95% confidence intervals (CI). The covariates for both Cox proportional hazard and multivariate logistic models were selected variables that had *p* < 0.1 in univariate comparisons between the groups. Univariate analyses were performed with SPSS software version 13. Multivariate analyses were conducted with SAS software version 9.1. The variables entered into the multivariate analyses included age, prior MI, stable angina, reference vessel diameter < 3 mm, lesion length, pre-procedural stenosis, type C, proximal and bifurcated lesions, angulated segments, drug-eluting stents, dissection and direct stenting.

## Results

As shown in Table 1, there were no significant differences in demographic characteristics of patients, including cardiovascular risk factors, age and history of PCI/coronary artery bypass grafting (CABG) despite the fact that these patients were not randomly assigned into the treatment groups. However, the number of patients with a history of MI was lower in the DS group (29.9 vs 35.7%, *p* = 0.01), while more patients in this group had presented with stable angina (51.1 vs 44.8%, *p* = 0.01).

**Table 1:** Clinical Characteristics Of Patients With Stenting After Pre-Dilatation Versus Direct Stenting

	*Direct group (n = 857) no (%)*	*Pre-dilatation group (n = 746) no (%)*	p*-value*
Age (years)*	55.8 ± 10.3	56.1 ± 10.7	0.52
Male	607 (70.8)	532 (71.3)	0.83
Diabetes mellitus	185 (22)	163 (22.1)	0.97
Positive family history	173 (20.2)	169 (22.7)	0.23
Hyperlipidaemia	367 (43.6)	324 (43.8)	0.91
Smoker	334 (39)	307 (41.2)	0.37
Hypertension	299 (35.5)	251 (34)	0.52
Prior PCI	54 (6.3)	57 (7.7)	0.29
Prior CABG	24 (2.8)	21 (2.8)	0.98
Prior MI*	256 (29.9)	266 (35.7)	0.01
Stable angina*	436 (51.1)	334 (44.8)	0.01
Ejection fraction < 40%	41 (6.7)	35 (6.5)	0.9

*Adjusted and fixed in multivariate analysis. PCI = percutaneous coronary intervention; CABG = coronary artery bypass grafting; MI = myocardial infarction.

## Angiographic and procedural data

Lesion characteristics are shown in Table 2. In the DS group, lesions were shorter in length, larger in diameter, and had lower pre-procedural diameter stenosis. Type C and diffuse lesions were less frequently seen in this group (11.9 vs 35.8% and 9.7 vs 30.2%, *p* < 0.001). Except for the higher prevalence of proximal lesions in the DS group, there were no further significant differences in the location of lesions in these two populations.

In 27 patients, the stents could not pass the lesion directly. These patients were allocated to the PS group without any stent dislodgement or other complications. Stent diameter and length ranges were 2.5–4 mm and 8–39 mm. In the DS group, stents were shorter with a larger diameter, and drug-eluting stents were less frequently used (Tables 2, 3). The stents were deployed at a mean inflation pressure of 12.9 ± 2.8 atm in the DS versus 12.9 ± 2.6 atm in the PS groups (*p* = 0.9).

**Table 2:** Lesion And Procedural Characteristics In Patients Treated With Direct Stenting Versus Stenting After Pre-Dilatation

	*Direct group (n = 857) no (%)*	*Pre-dilatation group (n = 746) no (%)*	p*-value*
Multivessel disease	296 (45.3)	287 (48.6)	0.24
LAD territory	522 (60.9)	444 (59.5)	0.57
RVD (< 3 mm)*	211 (24.6)	260 (34.9%)	< 0.001
Lesion length (mm) (mean ± SD)*	14.2 ± 6.01	19.4 ± 8.95	< 0.001
Pre-procedural stenosis (%)	85.7 ± 9.5	93.4 ± 6.3	< 0.001
Type C lesions*	96 (11.9)	242 (35.8)	< 0.001
Diffuse	83 (9.7)	225 (30.2)	< 0.001
Proximal lesion*	358 (41.8)	235 (31.5)	< 0.001
Angulated segments (> 45°)*	11 (1.3)	19 (2.5)	0.06
Bifurcation*	33 (3.9)	43 (5.8)	0.07

*Adjusted and fixed in multivariate analysis. RVD = reference vessel diameter; LAD = left anterior descending artery.

## In-hospital results

Despite similar angiographic success rates in both groups, the procedural success rate was significantly higher in the DS group (Table 3). Dissection occurred in two (0.2%) patients in the DS versus 29 (3.9%) in the PS groups (*p* < 0.001). In multivariate analysis, direct stenting was associated with a lower risk of dissection (OR = 0.07, 95% CI: 0.01–0.33, *p* < 0.001) after adjusting for significant covariates that are marked in Tables 2–4. Other factors that showed to be independent predictors for dissection were stable angina (OR = 3.38, 95% CI = 1.24–9.21, *p* = 0.017), type C lesions (OR = 5.13, 95% CI = 1.97–13.33, *p* < 0.001), angulated segments (OR = 7.09, 95% CI = 1.71–29.45, *p* = 0.007) and lesion length (OR = 0.94, 95% CI = 0.88–0.99, *p* = 0.04).

**Table 3:** Procedural Data And In-Hospital Outcomes In Patients Treated With Direct Stenting Versus Stenting After Pre-Dilatation (Univariate Analysis)

	*Direct group (n = 857) no (%)*	*Pre-dilatation group (n = 746) no (%)*	p*-value*
Drug-eluting stents*	99 (12)	131 (18.3)	0.001
Stent length (mm)*	16.09 ± 5.91	19.99 ± 6.56	< 0.001
Stent diameter (mm)*	3.08 ± 0.35	2.98 ± 0.36	< 0.001
Dissection*	2 (0.2)	29 (3.9)	< 0.001
Non-Q-wave MI	5 (0.6)	15 (2.1)	0.01
Angiographic success	854 (99.9)	740 (99.2)	0.06
Procedural success	848 (99.2)	725 (97.2)	0.002

*Adjusted and fixed in multivariate analysis. MI = myocardial infarction.

**Table 4:** Late Clinical Outcomes In Patients Treated With Direct Stenting Versus Stenting After Pre-Dilatation (Multivariate Analysis)

	*Direct group (n = 753) no (%)*	*Pre-dilatation group (n = 651) no (%)*	p*-value (univariate analysis)*	p*-value (multivariate analysis)*	*Adjusted hazard ratio (95% CI) for DS vs PS groups*
MACE	37 (4.9)	30 (4.6)	0.79	0.7	1.1 (0.58–2.11)
Cardiac death	4 (0.5)	5 (0.8)	0.74	0.64	0.57 (0.12–3.63)
Non-fatal MI	11 (1.5)	12 (1.8)	0.57	0.27	1.94 (0.6–6.22)
TVR	22 (2.9)	16 (2.5)	0.59	0.84	0.91 (0.38–2.17)
TLR	10 (1.3)	3 (0.46)	0.07	0.1	0.25 (0.05–1.33)
CABG	12 (1.6)	13 (2)	0.44	0.41	1.54 (0.54–4.35)

MACE = major adverse cardiac events; MI = myocardial infarction; TVR = target-vessel revascularisation; TLR = target-lesion revascularisation; CABG = coronary artery bypass grafting.

In each group, one patient (0.1%) developed unstable angina and one (0.1%) developed Q-wave MI in hospital. More patients in the PS group developed non-Q-wave MI in hospital (Table 4). In the multivariate logistic regression model, type C lesion as an independent factor was associated with increased risk of in-hospital non-Q-wave MI (OR = 5.62, 95% CI = 1.54–20.42, *p* = 0.008). However, stenting technique was not an independent predictor for the occurrence of non-Q-wave MI (OR for DS vs PS = 0.3, 95% CI = 0.06–1.3, *p* = 0.1).

## Long-term clinical outcomes

The mean follow-up times available for the patients who successfully survived the hospital period were 11.3 ± 4.7 months for the DS and 11.1 ± 4.9 months for the PS groups. There was no difference in MACE between the two groups with univariate analysis. After adjusting for selective variables that had significant differences between the groups, there was still no difference in the occurrence of MACE between the groups. However, reference vessel diameter (RVD) < 3 mm was found to be an independent predictor for MACE in the total population. The long-term adjusted hazard ratios for MACE and its constructing components in the DS versus PS groups are displayed in Table 4. MACE-free survival rates were also similar in both groups at 16 months’ follow-up (91.6% in patients treated with direct stenting vs 91.2% in those treated with stenting after pre-dilatation, *p* = 0.96).

## Discussion

## Long-term outcomes

**Fig. 1.**
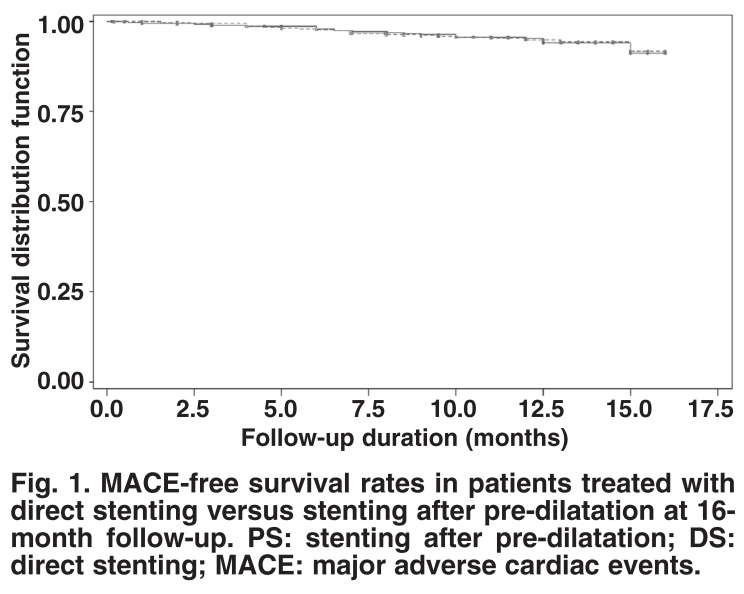
MACE-free survival rates in patients treated with direct stenting versus stenting after pre-dilatation at 16-month follow-up. PS: stenting after pre-dilatation; DS: direct stenting; MACE: major adverse cardiac events.

The main finding that emerged from this real-world cohort study was that long-term MACE and MACE-free survival after direct stenting was at least comparable to that for stenting after balloon pre-dilatation (Table 4, Fig. 1). The observations in experimental studies have allowed us to hypothesise that direct stenting might reduce the restenosis rate.^20^ This hypothesis is based on the idea that direct stenting may reduce the initial damage caused to the artery wall, as we know that pre-dilalation may cause dissection, and the magnitude of balloon-induced barotraumas could influence the rate of in-stent restenosis.^21^ Moreover, the beneficial effect of direct stenting in reducing the procedure duration, costs and radiation exposure has been proven in many studies.^8^-^10^,^22^,^23^ On the other hand, direct stenting could have some disadvantages that might increase the risk of restenosis because of the insufficient deployment of the stent, underestimation of the artery diameter or the lesion length, and difficulty with accurate stent placement.^24^

In the BET trial, it was shown that direct stenting had no influence on long-term TLR.^24^ The ISAR-DIRECT and PREDICT trials also showed that DS was not associated with reduction of thrombotic and restenotic complications, compared to PS.^25^,^26^ Likewise, the authors of another study showed that DS was not significantly associated with lower MACE rates.^13^ Our findings are consistent with the results of all these studies. Indeed, it seems that the probable advantages of direct stenting in reducing the restenosis rate remain a hypothesis, as they have not been clinically proven in any trials.

However, two of the limitations of some of these trials were that patients were mostly highly selected, or there was a small sample size. This means caution is required before making any accurate judgments about the outcomes of stenting with conventional or direct stenting methods.

In our study, RVD < 3 mm was an independent predictor for MACE in the total population. In a previous study on a population of consecutive patients over two years, we had shown that low ejection fraction and PCI on the left anterior descending artery were independent predictors for the occurrence of MACE.^27^

## In-hospital outcomes

As a secondary aim, we found out that in-hospital non-Q-wave MI was more prevalent in patients subjected to pre-dilatation, contributing to a lower procedural success rate. Moreover, dissection rate was higher in patients assigned to stenting after pre-dilatation.

Some studies have shown that DS is a feasible and safe procedure with immediate good primary success and low in-hospital complication rates in a wide range of clinical cases and lesion types.^28^ In addition, other studies have shown that direct stenting may be easily performed in complex multi-vessel, smaller-calibre or long or distal lesions^29^ in addition to non-complex and non-calcified lesions.^30^,^31^

With univariate analysis, we found higher procedural success rates and less frequent in-hospital non-Q-wave MI in patients treated with direct stenting. However, with the multivariate model, only complex lesions (type C) were independent predictors for in-hospital MI, and stenting techniques did not have an independent role for the occurrence of this event.

Many studies have similarly shown no difference in the rate of non-Q-wave MI between the patients treated with these two techniques.^7^,^8^, ^23^,^26^ On the other hand, a lower incidence of troponin release and non-Q-wave MI with DS compared to PS has been reported in some studies.^32^,^33^ The higher procedural success rate (due to lower frequency of non-Q-wave MI) in the DS group in our study may also be attributed to the lower frequency of type C lesions in this population. However, the influence of operator experience remains difficult to measure, particularly in the choice of guiding catheters and stents, in their manipulation, and in the lesion-crossing manoeuvre without pre-dilatation.^34^

With the multivariate logistic regression model, we found that direct stenting as an independent risk factor was associated with a lower incidence of dissections compared to stenting after pre-dilatation. This finding is consistent with reports in previous studies. The incidence of dissections after pre-dilatation has ranged from nine to 45% in previous studies.^19^,^35^ Some reports have suggested that direct stenting is associated with a reduced rate of dissections,^8^,^36^ particularly when thrombus is present or when treating ageing vein grafts.^37^ On the other hand, it has been reported that pre-dilatation may cause dissections that extend beyond the initial lesion.^38^ Our results are consistent with the results of all these studies.

It may be worthwhile noting the results of a meta-analysis of 10 randomised trials, which showed that DS compared with PS in selected coronary lesions was safe, optimised the use of equipment, and might enhance the early results of coronary intervention, while warranting similar late clinical outcomes.^39^

There were several limitations to our study. Firstly, follow-up information was not available in 12% of the patient cohort. However, we have no reason to believe that this would have affected one group more than the other, and would not therefore alter the conclusions in any significant way. Secondly, the study was not randomised and this may have jeopardised the conclusions to some extent. However, we adjusted the outcome data for differences in baseline characteristics, adding further credence to the conclusions.

## Conclusion

This study demonstrated that patients undergoing direct stenting in the real world had at least similar long-term outcomes to patients treated with stenting after pre-dilatation. Regarding acute procedural success and in-hospital events, our data showed lower rates of dissection and non-Q-wave MI in patients treated with the direct-stenting strategy. However, after statistical adjustments, direct stenting was only accompanied by reduced risk of dissection.
